# Reply to Orsi, M.; Somigliana, E. Incarceration of the Gravid Uterus: Proposal for a Shared Definition. Comment on “Tachibana et al. Incarcerated Gravid Uterus: Spontaneous Resolution Is Not Rare. *Diagnostics* 2021, *11*, 1544”

**DOI:** 10.3390/diagnostics12010030

**Published:** 2021-12-24

**Authors:** Daisuke Tachibana, Takuya Misugi, Kohei Kitada, Yasushi Kurihara, Mie Tahara, Akihiro Hamuro, Akemi Nakano, Akira Yamamoto, Masayasu Koyama

**Affiliations:** 1 Department of Obstetrics and Gynecology, Osaka City University Graduate School of Medicine, Osaka 545-8585, Japan; misutaku1975@infoseek.jp (T.M.); kafukafu0404@yahoo.co.jp (K.K.); kurikuri_1011@yahoo.co.jp (Y.K.); rxv13436@nifty.ne.jp (M.T.); hamuroa@med.osaka-cu.ac.jp (A.H.); m2037746@med.osaka-cu.ac.jp (A.N.); masayasukoyama@gmail.com (M.K.); 2 Department of Diagnostic and Interventional Radiology, Osaka City University Graduate School of Medicine, Osaka 545-8585, Japan; loveakirayamamoto@gmail.com

We appreciate the interest in our paper, and we are grateful for the comment by Orsi et al., ‘Incarceration of the gravid uterus: proposal for a shared definition’. They proposed that both symptomatic cases at any gestational age and asymptomatic full-term cases should be included in the definition.

However, symptoms in early trimesters might be caused not only by incarceration itself, but also by the retroflexed and/or retroverted uterus. Moreover, the paramount message of our paper was that awareness and enlightenment for the sustained incarceration should be emphasized, because this condition may lead to severe perinatal outcomes [1]. We consider that the choice of 16 weeks of gestation as an inclusion threshold is reasonable.

## Data Availability

Data available on request due to restrictions of privacy.
